# Ovarian ectopic pregnancy: A rare case

**Published:** 2014-04

**Authors:** Hatav Ghasemi Tehrani, Zaynab Hamoush, Mojdeh Ghasemi, Leila Hashemi

**Affiliations:** 1 *Department of Obstetrics and Gynecology, Shahid Beheshti Hospital, Isfahan University of Medical Sciences, Isfahan, Iran.*; 2 *Research Office of Shahid Beheshti Hospital, Isfahan University of Medical Sciences, Isfahan, Iran.*

**Keywords:** *Ovarian pregnancy*, *Intra uterine devices*, *Laparotomy*, *Pathology*

## Abstract

**Background:** Ovarian pregnancy is a rare form of the non-tubal ectopic pregnancy. It ends with rupture before the end of the first trimester. One of the important risk factors for ovarian pregnancy is in the use of Intra uterine devices (IUD).

**Case: **We report here one such uncommon case of ovarian ectopic pregnancy. Our patient is a 30 years old multiparous woman with two previous cesarean sections with severe hypogastric abdominal pain. During laparotomy, ruptured ovarian ectopic pregnancy was diagnosed, and wedge resection of the ovary was only done. Histopathological examination confirmed it to be an ovarian ectopic pregnancy.

**Conclusion:** IUD is one of contraceptive methods which prevents intra-uterine implantation in 99.5%, if implant occurs with IUD, it is tubal implantation in 95% of cases, and it is very rare in other places such as ovary. The most important risk factor of ovarian ectopic pregnancy is IUD as in this study it was showed.

## Introduction

Ovarian ectopic pregnancy is a rare variant of ectopic implantation (1). It ends with rupture before the end of the first trimester (2). Ovarian ectopic pregnancy incidence after natural conception ranges from 1 in 2000 to 1 in 60 000 deliveries and accounts for 3% of all ectopic pregnancies (3, 4). One in every nine ectopic pregnancies among Intra uterine devices (IUD) users is an ovarian pregnancy (5, 6). The diagnosis is intricate and based on surgical and histopathological observations (3). 

Ectopic pregnancy implanted in the ovary is rare. Traditional risk factors for ovarian ectopic pregnancy are similar to those for tubal pregnancy, but use of an IUD seems to be disproportionately associated. Although the ovary can accommodate more readily than the fallopian tube to the expanding pregnancy, rupture at an early stage is the usual consequence (7).

**Diagnosis**

Findings are likely to mimic those of a tubal pregnancy or a bleeding corpus luteum. Serious bleeding is seen in approximately one third of cases. At surgery, early ovarian pregnancies are likely to be considered corpus luteum cyst or a bleeding corpus luteum. Use of transvaginal sonography has resulted in a more frequent diagnosis of unruptured ovarian pregnancies (7).

**Management**

The classical management for ovarian pregnancies has been surgical. Early bleeding for small lesion has been managed by ovarian wedge resection or cystectomy. With larger lesions ovariectomy is most often performed and laparoscopy has been used to resect or to perform laser ablation. Finally methotrexate has been used successfully to treat unruptured ovarian.

## Case report

Our patient is a 30-year-old multiparous woman with two previous cesarean sections due to twin pregnancy, and one time Dilatation and curretage (D&C) for spontaneous abortion. For writing this study, oral consent was obtained from our case. She admitted with severe abdominal pain in hypogastric area. Pain was continues and radiated to left shoulder. Her last menstrual period (LMP) was 2 months ago and her menstruation cycle was regular. 

She had the history of using IUCD (copper) since her last delivery. Because of her menstruation retardation, she checked βhCG, that was positive, she didn’t take sonography before admitting in hospital. The βhCG titer during hospitalization shows only a slow rise in the tittering in plateau form. The patient has no previous history of any medical condition, and she was hemodynamically stable. Transvaginal sonography was done for the first initial impression of an ectopic pregnancy; TVS showed a uterus of normal size with no gestational sac, but an ovarian cyst 11×11mm in the right side with another echogenic mass medially to the right ovary of 9×11 mm size and right tubal ectopic pregnancy was suggested. The first treatment plan of this patient was close observation and multiple dose of methotrexate since the patient is hemodynamically stable.

During medical treatment βhCG titer didn’t decrease and the patient suffered from a severe abdominal pain and her vital signs were unstable; BP= 80/P, Pulse rate=120, and patient was pail. Laparotomy was suggested. During laparatomy, there was about 500-600 CC blood in the abdomen cavity, and ruptured ovarian ectopic pregnancy was diagnosed. Suction of blood and wedge Resection of the ovary was performed. Histopathological examination confirmed an ovarian ectopic pregnancy.

## Discussion

An ectopic pregnancy is characterized by implantation and development of an embryo outside of the uterine cavity. Ectopic pregnancies can occur in the ovary (3.2%), or abdomen (1.3%) (8). Hertig estimated that ovarian pregnancy occurs in one in 25 000 to 40 000 pregnancies (9). It is characterized by a poor clinical symptomatology and a difficult ultrasound diagnosis. The surgical criteria remain hard to prove (10). Intrauterine contraceptive devices may also be a cause (11). As a matter of fact, an intrauterine contraceptive device is found in 14-30% of patients with a nonovarian extra uterine pregnancy while it is found in proportions ranging from 57-90% of patients with a primary ovarian pregnancy (12-18). 

Its action could be explained by altered tubal motility, thereby facilitating the implantation in the ovary (10). The increase in the incidence of ovarian pregnancy is closely related to the use of intra uterine devices (IUD) as a contraceptive method. These prevent uterine implantation, but do not provide protection against ovarian implantation (19). A study showed the strong association of IUD as a risk factor of ovarian pregnancy thus IUD reduces uterine implantation by 99.5%, tubal implantation in 95% and they have no effect on ovarian location (19-21).

The lowest ectopic pregnancy rates is seen for the most effective IUD like TCu380 A (1). Also, fertility treatment had remained important associated risk factor (20). Patients mainly have symptoms as in ectopic pregnancy at other sites (1). Ovarian pregnancy can be missed diagnosed with ruptured corpus luteum cyst in 75% of cases (21, 22). Chronic pelvic pain alone is the most frequent clinical symptoms of an ovarian gestation as in our patient, also an adnexal mass may be palpable on examination (22-23). The diagnosis is often made at surgery and requires histological confirmation. A correct diagnosis of ovarian pregnancy during surgery is only possible in 28% of the cases, because it is difficult to differentiate from a hemorrhagic corpus luteum intraoperatively (19). 

Diagnosis is based on the classic description of a cyst with a wide ectogenic outer ring using ultrasound (20, 21). Patel *et al* reported a rare case of twin ovarian pregnancy diagnosed by ultrasound (24). Although Ultrasound may suggest the diagnosis, surgery (laparoscopy or laparotomy) remains the best method of a differential diagnosis and management (22, 25, 26). 

A high index of suspicion is based upon a combination of ultrasound finding, (both gray scale and color Doppler), as well as high levels of serum HCG and sonographic experience (22). Ultrasound diagnosis of an ovarian ectopic pregnancy may be different such as serum cell tumors or other ovarian pathology (22-25). Diagnosis is suspected during laparoscopy or laparatomy and confirmed by histopathology (24-27). Treatment of almost all known ovarian ectopic pregnancies has been surgical. Also case reports have described successful MTX therapy (1). 

In our case, since the patient was hemodynamically stable and HCG titer was in plateau, we suggested multiple dose of MTX treatment but when the patient suffered from severe abdominal pain and tenderness of the right lower quadrant, we suggested laparatomy. Patel *et al* showed that the most surgical treatment of ovarian ectopic pregnancy consists on wedge resection and oophorectomy (24, 28). During our laparatomy, an ovarian pregnancy was clearly seen and an ovarian wedge resection was done. Resta et al reported a case of ruptured ovarian ectopic pregnancy despite low levels of beta HCG. In our case there were no rupture and HCG was slowly raised (29). Histopathologist confirmed an ovarian ectopic pregnancy. Ovarian ectopic pregnancy is a rare variant of ectopic gestation. The diagnosis is made often at surgery and requires histologic confirmation. For a woman with a previous history of ectopic pregnancy, it is better not to use a copper or levonorgestrol containing IUD.
